# The World‐Wide FINGERS Global Strategy for Precision Prevention of Dementia and Alzheimer’s Disease

**DOI:** 10.1002/alz.093013

**Published:** 2025-01-09

**Authors:** Francesca Mangialasche, Anna Matton, Tiia Ngandu, Jenni Lehtisalo, Chi Udeh‐Momoh, Vilma Alanko, Mariagnese Barbera, Celeste A de Jager, Markku Peltonen, Dinithi Perera, Ruth Stephen, Alina Solomon, Miia Kivipelto, World‐Wide FINGERS network

**Affiliations:** ^1^ Division of Clinical Geriatrics, Center for Alzheimer Research, Department of Neurobiology, Care Sciences and Society, Karolinska Institutet, Stockholm Sweden; ^2^ Karolinska University Hospital, Theme Inflammation and Aging, Solna Sweden; ^3^ FINGERS Brain Health Institute, Stockholm Sweden; ^4^ Finnish Institute for Health and Welfare, Helsinki Finland; ^5^ Division of Clinical Geriatrics, Center for Alzheimer Research, Department of Neurobiology, Care Sciences and Society (NVS), Karolinska Institute, Stockholm Sweden; ^6^ Department of Clinical Medicine/Neurology, University of Eastern Finland, Kuopio, Finland, Kuopio Finland; ^7^ Wake Forest University School of Medicine, Salem, NC USA; ^8^ Brain and Mind Institute, Aga Khan University, Nairobi Kenya; ^9^ Division of Clinical Geriatrics, Karolinska Institutet, Stockholm Sweden; ^10^ Division of Clinical Geriatrics, Center for Alzheimer Research, Department of Neurobiology, Care Sciences and Society (NVS), Karolinska Institutet, Stockholm Sweden; ^11^ The Ageing Epidemiology Research Unit, School of Public Health, Imperial College London, London United Kingdom; ^12^ University of Eastern Finland, Kuopio Finland; ^13^ The Ageing Epidemiology (AGE) Research Unit, School of Public Health, Imperial College London, London United Kingdom; ^14^ Division of Clinical Geriatrics, Alzheimer Research Center, Karolinska Universitetssjukhuset, Solna, Stockholm Sweden; ^15^ FINGERS Brain Health Institute, Stockolm, Stockholm Sweden; ^16^ Division of Clinical Geriatric (NVS), Karolinska Institutet, Sweden, Stockholm Sweden; ^17^ Department of Neurobiology, Care Sciences and Society, Center for Alzheimer Research, Division of Clinical Geriatrics, Karolinska Institutet, Stockholm Sweden

## Abstract

**Background:**

Precision Prevention –the right intervention for the right person at the right time– is a recent endeavour in the Alzheimer’s disease (AD) field, requiring the integration of multidimensional data from large, representative populations, to identify phenotypes for early and accurate detection of prevention potential.

**Aims:**

The World‐Wide FINGERS (WW‐FINGERS) network of multidomain trials for risk reduction and prevention of dementia includes 60+ countries. The network aims to adapt and test the original model from the Finnish Geriatric Intervention Study to Prevent Cognitive Impairment and Disability (FINGER) which showed that a multidomain intervention can benefit cognition in older adults at‐risk of dementia. WW‐FINGERS works towards trials data harmonization, to enable joint data analysis.

**Methods:**

International Working Groups (IWGs) focused on trials´ prominent features were established to leverage the WW‐FINGERS expertise, facilitate data harmonization and joint analysis. Mapping and harmonization of data across the trials is ongoing, via periodic meetings and RedCap‐supported digital surveys done within the network.

**Results:**

The WW‐FINGERS network completed 14 trials, while 21 are ongoing, accounting for 18000+ participants. Trial Harmonization Guidelines were compiled, to support prospective data harmonization. Two IWGs –Cognitive Outcomes and Biorepository/Biomarkers– conducted surveys to map the current data and sample collection within the network. The Cognitive Outcome survey included data from 25 studies, showing target population ‐from asymptomatic at‐risk of dementia to early symptomatic stages‐, ethnicities within the trials (including 20% Caucasian, 10% Asian, 8% Black African, 7.5% mixed ethnicity, 4% Hispanic/Latino, 5% Caribbean), use of cognitive tests in 19 languages, with composite neuropsychological test batteries being most frequently used, capturing memory, executive function, and processing speed. The Biorepository/Biomarker survey was completed for 29 trials, including information on sample types, processing, storage, *APOE* availability and other biomarkers. Survey results are informing data harmonization and protocols for new trials. Additional IWGs are planned, including Neuroimaging, Non‐Cognitive outcomes and Health‐economics. Collaborations with international stakeholders are ongoing to maximize outreach and enable data sharing.

**Conclusion:**

The WW‐FINGERS global collaboration is enabling scalability and evolution of the FINGER model, and prospective harmonization of multidomain trials, establishing the base for a Precision Prevention approach in AD and dementia.

